# Supplementation of black soldier fly larvae (*Hermetia illucens*) as a sustainable protein source on growth performance, blood profiles, immune response, and diarrhea incidence in weaning pigs

**DOI:** 10.5713/ab.25.0425

**Published:** 2025-09-30

**Authors:** Sooduc Noh, Xinghao Jin, Minhyuk Jang, Minsoo Park, Yooyong Kim

**Affiliations:** 1 Department of Agricultural Biotechnology and Research Institute of Agriculture and Life Sciences, Seoul National University, Seoul, Korea; 2 College of Agriculture, Yanbian University, Yanji, China

**Keywords:** Black Soldier Fly Larvae, Diarrhea Incidence, Growth Performance, Immune Response, Weaning Pigs

## Abstract

**Objective:**

This study investigates the feasibility of substituting fishmeal with black soldier fly larvae (BSF) in weaning pig diets, with the objective of overcome the limitations of conventional animal protein sources and explore BSF larvae as a sustainable alternative protein.

**Methods:**

A total of 192 weaning ([Yorkshire×Landrace])×Duroc) pigs (8.12±0.01 kg body weight [BW]) were assigned to four treatments based on sex and initial BW, with six replicates of eight pigs per pen in a randomized complete block design. Experimental diets with different levels of BSF larvae were as follows: 1) Control: corn–soybean-based diet, 2) BSF25: corn–soybean-based diet containing black solder fly larvae as a replacement for 25% of plasma protein, 3) BSF50: corn–soybean-based diet containing black solder fly larvae as a replacement for 50% of plasma protein, 4) BSF100: corn–soybean-based diet containing black solder fly larvae as a replacement for 100% of plasma protein.

**Results:**

From 0–2 weeks, the average daily gain and gain:feed ratio were decreased as the BSF larvae level increased (p<0.05), and the BSF25 group had higher BW compared to other groups (p<0.05). In blood profiles, increasing levels of BSF larvae in the diet linearly decreased albumin levels at 28 d after weaning (p<0.05). For immune response, there was a linear decrease in IgG concentration by increasing levels of BSF larvae (p<0.05), and the BSF25 group had the highest value among all treatments only in phase I (p<0.05).

**Conclusion:**

The results of this study demonstrate that BSF larvae meal can be a viable alternative protein source in diets of weaned pigs. The optimal ratio of BSF larvae replacing plasma protein is up to 25%, which leads to improved growth performance and immune response.

## INTRODUCTION

The global population increase and the development of meat consumption cultures may significantly impact animal feed use and land demand [1]. A recent report shows that pork consumption accounted for 24% of global meat consumption in 2023, and poultry and beef took 40% and 22%, respectively [2]. For the world’s pig industry, in 2023, almost 1.3 billion pigs will be produced, and the production of pig feed is predicted to be 323 million metric tons [3]. To meet the continuously increasing demand for pig feed, the Food and Agriculture Organization suggested finding alternative raw materials to conventional feed ingredients for a stable feed supply [4]. Animal proteins (fish meal, plasma protein [PPR], etc.) are the most costly and have been used extensively in pig diet formulas. However, these conventional animal protein sources will intensify competition between human and animal feed chains [5]. Therefore, developing new, sustainable, high-quality protein sources is a crucial challenge for the pig industry.

With an estimated 1,900 species of edible insects, including the black soldier fly (BSF) larvae, the future of sustainable protein sources for animal feed looks promising [6]. The nutritional value of BSF larvae, rich in protein, lipids, vitamins, and minerals, has been well-documented [7]. The defatted processing of BSF larvae products used in swine research is classified into three types: full-fat BSF larvae, partially defatted BSF larvae, and defatted BSF larvae, with the primary difference being in fat content [8]. Defatted and full-fat BSF larvae boast a rich amino acid profile, particularly leucine, lysine, and valine [9]. Unlike other insects, BSF larvae can be reared easily on several organic wastes, with a higher feed conversion rate, survival rate, and rearing costs [10]. Given these nutritional benefits, BSF larvae present a promising alternative protein source that could help address the global food challenges.

While several studies have evaluated the inclusion of BSF larvae in weaning diets, particularly in terms of growth performance and nutrient digestibility [11] and modifications to blood profiles [12], as well as potential gut health implications [13,14], the majority of these studies have focused on replacing soybean meal. However, the effects of BSF larvae replacing animal protein sources in diet formulas remain unexplored, highlighting the need for more research.

Therefore, the present study aimed to investigate the effects of BSF larvae replacing PPR on growth performance, blood profiles, immune response, and diarrhea incidence in weaning pigs.

## MATERIAL AND METHODS

### Experimental animals and management

A total of 192 weaning ([Yorkshire×Landrace])×Duroc) pigs (8.12±0.01 kg body weight [BW]) were allotted to one of four treatments based on sex and initial BW, with six replicates of eight pigs per pen in a randomized complete block design. All pigs were housed in slotted plastic floor pens equipped with a feeder and a nipple waterer and allowed *ad-libitum* access to feed and water throughout the experimental period (Phase I: 0–2 weeks and Phase II: 3–4 weeks). The temperature in the experimental house was maintained at 30°C in the first week, decreased by 1°C every week, and was 27°C in the last week.

### Experimental design and diet

The BSF larvae replacing PPR used in this study were provided by Foodyworm. The nutritional composition of the BSF larvae meal and PPR is presented in Table 1. The treatments for early and late weaning phases were as follows: 1) Control: corn-soybean-based diet, 2) BSF25: corn-soybean-based diet with 25% of PPR replaced by BSF larvae, 3) BSF50: corn-soybean-based diet with 50% of PPR replaced by BSF larvae, 4) BSF100: corn-soybean-based diet with 25% of PPR replaced by BSF larvae. All nutrients in the experimental diets were formulated to meet or exceed the NRC [15] requirements. The formulation of the experimental diets was presented in Tables 2, 3.

### Growth performance

BW and feed intake data were collected at the end of each phase to calculate average daily gain (ADG), average daily feed intake (ADFI), and the gain to feed (G:F) ratio. In addition, the amount of feed eaten by all piglets was recorded each day, and waste feed in the feeder was recorded at the end of each phase.

### Blood sampling and analyses

Blood samples were taken from the jugular vein of twelve pigs with nearly average BWs in each treatment after 3 h of fasting to measure albumin (ALB), creatinine (CRE), glucose, total protein, globulin, urea, types of cholesterol, and immune response (IgA, IgG) when BW was recorded. The blood samples were centrifuged for 15 min at 1,207×g and 4°C (centrifuge 5810R; Eppendorf). The sera were transferred to 1.5 mL plastic tubes (BD vacutainer SSTTMII advance; Becton-Dickinson) and stored at −20°C until analysis. Serum IgG and IgA concentrations were analyzed by ELISA assay by the manufacturer’s protocols (ELISA Starter 87 Accessory Package, Pig IgG ELISA Quantitation Kit, Pig IgA ELISA Quantitation Kit; Bethyl). Total protein concentration (modular analytics, PE; Roche) and glucose (enzymatic kinetic assay; Roche) concentrations were analyzed using a blood analyzer. Total cholesterol (TC), high-density lipoprotein (HDL), low-density lipoprotein (LDL), ALB, and CRE were measured using spectrophotometric kits following the manufacturer’s instructions (TBA-120FR; Toshiba Medical Systems Corporation). Globulin was analyzed with the automatic analyst Olimpus AU 600 (Diagnostica).

### Incidence of diarrhea

The diarrhea incidence was determined at 8:00 am for 28 days. Data were recorded by pen during phases 1 and 2. The incidence of diarrhea was scored on a 4-point scale according to the condition of the feces and diarrhea (0 = normal feces; 1 = moist feces; 2 = mild diarrhea; 3 = severe and watery diarrhea). Slightly wet feces on the rump area was designated as contaminated. After recording these data, the watery diarrhea was cleaned away.

### Statistical and chemical analyses

All the collected data were carried out using least squares mean comparisons and evaluated using SAS’s general linear model procedure [16]. Each pen was used as a single unit in the feeding trial, and individual pigs were used as experimental units for blood profiles, immune response, and incidence of diarrhea. Orthogonal polynomial contrasts were performed to determine linear and quadratic effects of inclusion levels of BSF larvae. A one-way ANOVA analysis was performed to compare the control to other treatments. Statistical differences were considered highly significant differences at p<0.01, significant differences at p<0.05, and tendencies between p≥0.05 and p≤0.10.

## RESULTS

### Growth performance

The effects of BSF larvae replacing PPR on growth performance are presented in Table 4. In phase I, the ADG and G:F ratio decreased when the BSF larvae level increased (p<0.05; p≤0.05). In comparison with other treatments, the highest BW was observed in the BSF25 group (p<0.05). During all phases, a decreased trend of ADG was shown as the BSF larvae level increased (p≤0.1). However, the final BW (4 weeks) from all treatments did not show any differences.

### Blood profiles

The effects of BSF larvae levels on blood profiles are presented in Table 5. In phase I, the pigs from the control and BSF100 groups showed a higher trend of HDL cholesterol levels than those from the BSF25 and BSF50 groups (p≤0.1). In phase II, ALB level decreased as BSF larvae level increased (p<0.05), and pigs from the control and BSF25 treatments had higher values in ALB levels than BSF50 and BSF100 groups (p<0.05). During all phases, there were no significant differences in CRE, glucose, total protein, globulin, urea, or TC levels.

### Immune response

The effects of BSF larvae replacing PPR on immune response are presented in Table 6. The study found no significant difference in IgA when pigs were fed BSF larvae meal during the experimental period. However, in phase II, the IgG concentration in the BSF25 group showed a higher trend than other groups (p≤0.1), and then a quadratic trend was also observed as the amount of BSF larvae replacing PPR increased (p≤0.1).

### Diarrhea incidence

The effects of BSF larvae replacing PPR on the incidence of diarrhea are presented in Table 7. The change of diarrhea rate was not observed during the whole experimental period.

## DISCUSSION

According to previous studies, the BSF larvae used in swine diets can be divided into three types, which depend on the fat content [8]. Full-fat BSF larvae contain 35.9%–48.1% of crude protein (CP) and 36.8%–48.1% of crude fat (CF) [17]. An average of 59% CP and 9% CF was found in the partially defatted BSF larvae meal [18], and defatted BSF larvae meal showed the CP content ranged from 40.8%–60.7% and the CF content ranged from 7.97%–12.28% [19]. This study provided defatted BSF larvae meal, which possessed 59.34% of CP, 4.83% of CF, and 11.19% of crude fiber content. These nutritional differences may be related to the growth phase of BSF larvae, harvest point, and defatted-processing technology of BSF larvae meal [20,21]. Compared to PPR, BSF larvae had a poor amino acid profile, especially most of the essential amino acids related to the growth of weaning pigs (arginine, lysine, and threonine). Therefore, amino acid balance and or indispensable amino acid: CP ratios must be considered in diet formulation when adding BSF larvae meal to replace the PPR source fully. On the other hand, BSF larvae contained 8.42% chitin as potential functional constituents, which may positively enhance the health of monogastric animals [22,23]. If BSF larvae products achieve a degree of reliability in large-scale production, they will have great potential to partially replace animal protein sources in animal feeds.

In recent years, the supplementation of edible insects replacing traditional protein sources in animal diets has drawn more and more attention [9,24], which mainly focused on the inclusion levels that affect the growth performance in weaning pigs [18,25]. In the present study, during phase I, replacing 25% of PPR with BSF larvae improved the BW, but the ADG and G:F ratio linearly decreased as the ratio of BSFL replacing PPR increased. A similar result was obtained by Liu et al [14], who reported that a small quantity of BSF larvae supplemented (replacing 25% of soybean meal) could improve BW, and ADG and G: F ratio was decreased as the replaced amount exceeded 25% from weaned day 1 to 14. Spranghers et al [11] found that the addition of full-fat (4% and 8%) and defatted BSF (5.4) prepupae to diets had no significant differences in daily gain, feed intake, and feed-to-gain ratio compared to the control diet. Crosbie et al [26] also reported that 25% and 50% of the animal protein sources replaced by full-fat BSF larvae in weaning diets had no significant changes in BW, ADG, and ADFI overall in the weaning period. Ipema et al [27] found that supplementing with BSF larvae did not affect piglets’ ADG or final BW, nor did it impact their feed efficiency or energy efficiency. In this study, we found that adding BSF larvae replacing PPR had no detrimental effect on BW, ADG, ADFI, and G:F ratio at the final phase. The reasons for the growth differences may be related to the life stage of BSF larvae, rearing conditions, the replacement ratio, or fat content in BSF larvae [19]. Secondly, the total chitin content in the experimental diet could also affect growth performance. Chitin, a nitrogen-containing fiber that is relatively difficult to digest, may decrease the growth rate of piglets fed by BSF larvae due to an immature digestive system after weaning [14].

Limited information is available about the change in blood profiles when pigs are fed different BSF larvae levels. The current study observed only statistically significant differences in ALB and HDL concentrations at phase I or II. ALB regulates blood and body fluid volume through osmoregulation, accounting for over half of serum protein [28]. The normal serum ALB level in piglets is between 2–4 g/dL [29], and ALB levels are used to assess liver function, inflammation, and malnutrition [30]. Our study showed no significant differences in haematochemical parameters, except for the ALB concentrations, which showed that the control and BSF25 groups had the same statistical value and were higher than those in the BSF50 and BSF100 groups. This finding was in agreement with a study by Biasato et al [18], who reported that the addition of BSF larvae at 0%, 5%, and 10% to the diet did not result in significant differences in levels of globulin, CRE, total protein, or TC. Moreover, HDL cholesterol concentrations increased as pigs were fed with increased BSF larvae, replacing PPR. HDL cholesterol is considered the “positive” form of cholesterol, and higher levels within the normal range are associated with beneficial effects on cardiovascular health [31]. Chia et al [32] reported that serum lipid parameters, such as HDL and LDL concentrations, did not change among pigs fed with different levels of BSF larvae. However, in the present study, as the inclusion level of BSF larvae increased, HDL cholesterol levels also increased, suggesting an improvement in the blood lipid profile of piglets. This beneficial effect may be correlated with the change in intestinal microbiota due to the abundance of *subdoligranulum* [14]. Van Hul et al [33] reported that *subdoligranulum* was positively correlated with HDL levels in the blood.

IgG and IgA mainly represent the immune status of the body [34]. IgG is the most critical immunoglobulin in humoral immunity to prevent the invasion of infections or harmful bacteria. IgA is the body’s most common and secreted immunoglobulin, prohibiting the adhesion and colonization of pathogens in the intestines. In the current study, BSF25 groups had higher serum IgG concentrations than other groups in phase I, which contributed to improved pig performance, as shown in Table 4. The improvement of the immune response might be related to the chitin contained in BSF larvae. As an active compound, chitin has a positive effect on the immune response [35]. However, weaning pigs do not fully digested high levels of chitin to produce chitosan [36] and possess anti-nutritional properties [37]. In our study, the total chitin intake for each treatment can be calculated as follows: Control group, 0 mg; BSF25 group, 0.75 mg; BSF50 group, 1.46 mg; BSF100 group, 2.88 mg. Yousef et al [38] reported that the level of chitin exceeding the critical value harmed the immune response. Meanwhile, a study by Liu et al [14] also reported that BSF larvae as a replacement for SBM at 25% had higher IgM and IgG concentrations in the ileum of weaning pigs compared with the control or BSF larvae replacing 100% SBM.

Numerous studies have reported that incorporating insect protein into weaning pig diets can improve intestinal health, thereby reducing diarrhea [39,40]. Boontiam et al [39] reported that 12% of BSF larvae decreased diarrhea rate compared to the 6% of BSF larvae in weaning diets because the higher BSF-derived chitin (0.26% to 0.51%) may inhibit the growth of *Escherichia coli* and promote *Lactobacillus* spp. in the gut. Jin et al [40] also confirmed that BSF larvae supplementation of up to 8% can change gut microbiota distribution, increase lactobacillus’ relative abundance, and improve intestinal health. However, in the current study, there were no significant differences in diarrhea rates among the treatments. There was an agreement with the study by Liu et al [14], who reported that the rate of diarrhea in piglets from all treatments did not show any differences as the ratio of BSF larvae replacing SBM increased. Phaengphairee et al [41] also found that the incidence of diarrhea was unaffected by BSF larvae supplemented up to 12% in weaning pig diets compared to the control. This result predicted that weaning pigs could not secrete enough chitinase to produce chitooligosaccharides as prebiotics to improve gut health.

## CONCLUSION

In the current study, replacing PPR up to 25% with BSF larvae in weaning diets had a positive effects on growth performance, blood profiles, and immune response; BW or IgG concentration was especially improved in the early (0–2 weeks) or late weaning phase (4 week). However, the ratio of BSF larvae replacing protein plasma exceeded 50%, negatively affecting ADG and the G:F ratio. Therefore, it is possible to substitute up to 25% PPR by BSF larvae in the diet of weaning pigs.

## Figures and Tables

**Table 1:** Nutritional composition of black soldier fly (BSF) larvae and plasma protein (dry matter, 100%)

Nutrients	BSF larvae meal	Plasma protein
Crude protein (%)	60.57	84.67
Crude fat (%)	4.93	-
Ash (%)	19.98	8.86
Crude fiber (%)	11.42	0.06
Chitin (%)	8.60	-
Calcium (%)	6.63	0.15
Phosphorus (%)	1.00	1.68
Amino acid profile (%)		
Alanine	3.51	3.88
Arginine	2.87	4.37
Glycine	3.13	2.61
Histidine	1.77	2.39
Isoleucine	2.38	2.64
Leucine	4.02	7.38
Lysine	3.45	6.33
Phenylalanine	2.34	4.21
Proline	3.25	4.11
Serine	2.45	4.16
Threonine	2.25	4.26
Cystine	0.38	2.53
Methionine	0.96	0.62

Lab analysis values.

**Table 2:** Formulation of the experimental diets during the early weaning phase (0–2 wk)

Items	Treatment^1)^			

	Control	BSF25	BSF50	BSF100
Ingredient (%)				
Expanded corn	66.37	66.23	65.91	65.52
Soybean meal	9.40	9.20	9.00	8.50
Whey	10.00	10.00	10.00	10.00
Soy oil	3.00	2.80	2.70	2.40
Plasma protein	4.00	3.00	2.00	0.00
Fish meal	4.00	4.00	4.00	4.00
Black soldier fly larvae	0.00	1.50	3.00	6.00
L-Lysine, 98%	0.42	0.45	0.47	0.53
DL-Met, 98%	0.24	0.23	0.22	0.21
L-Threonine, 98.5%	0.18	0.20	0.21	0.25
Monocalcium phosphate	1.40	1.40	1.50	1.60
Limestone	0.00	0.00	0.00	0.00
Vit. Mix^2)^	0.12	0.12	0.12	0.12
Min. Mix^3)^	0.12	0.12	0.12	0.12
Salt	0.50	0.50	0.50	0.50
Zinc oxide	0.25	0.25	0.25	0.25
Sum	100.00	100.00	100.00	100.00
Chemical composition^4)^				
Metabolizable energy (kcal/kg)	3,517.00	3,516.00	3,516	3,515.00
Crude protein (%)	17.60	17.60	17.57	17.51
Crude fat (%)	6.26	6.28	6.38	6.49
SID lysine (%)	1.37	1.37	1.37	1.37
SID methionine (%)	0.52	0.52	0.51	0.51
SID threonine (%)	0.90	0.90	0.89	0.89
Total calcium (%)	0.58	0.61	0.66	0.73
Total phosphorus (%)	0.79	0.79	0.80	0.80
Chitin (%)^5)^	0.00	0.13	0.25	0.51

1) Control: corn-soybean meal-based diet, BSF25: corn-soybean meal based diet with 25% of plasma protein replaced by black soldier fly larvae, BSF50: corn-soybean meal based diet with 50% of plasma protein replaced by black soldier fly larvae, BSF100: corn-soybean meal based diet with 100% of plasma protein replaced by black soldier fly larvae.

2) Contents of vitamins provided per kg of complete diet: vitamin A, 10,000 IU; vitamin D3, 2,000 IU; vitamin E, 60 IU; vitamin K, 3.5 mg; vitamin B2, 8 mg; vitamin B6, 2 mg; vitamin B12, 35 μg; pantothenic acid, 25 mg; biotin, 100 μg; niacin, 50 mg; folic acid 3.1 mg; thiamine, 1.5 mg.

3) Contents of minerals provided per kg of complete diet: Fe, 100 mg; Mn, 50 mg; Zn, 50 mg; Cu, 80 mg; Se, 400 μg; I, 1 mg.

4) Lab analysis values.

5) Calculated values based on the chitin content of BSF (8.42%).

**Table 3:** Formula of the experimental diets during the late weaning phase (wk 3–^4)^

Items	Treatment^1)^			

	Control	BSF25	BSF50	BSF100
Ingredient (%)				
Expanded corn	63.77	63.59	63.40	63.24
Soybean meal	15.80	15.80	15.90	15.80
Whey	9.00	9.00	9.00	9.00
Soy oil	2.50	2.40	2.30	2.10
Plasma protein	2.00	1.50	1.00	0.00
Fish meal	4.00	4.00	4.00	4.00
Black soldier fly larvae	0.00	0.75	1.50	3.00
L-Lysine, 98%	0.49	0.49	0.50	0.52
DL-Met, 98%	0.30	0.30	0.30	0.30
L-Threonine, 98.5%	0.22	0.23	0.24	0.26
Monocalcium phosphate	0.55	0.57	0.59	0.61
Limestone	0.40	0.40	0.30	0.20
Vit. Mix^2)^	0.12	0.12	0.12	0.12
Min. Mix^3)^	0.10	0.10	0.10	0.10
Salt	0.50	0.50	0.50	0.50
Zinc Oxide	0.25	0.25	0.25	0.25
Sum	100.00	100.00	100.00	100.00
Chemical composition^4)^				
Metabolizable energy (kcal/kg)	3,480.00	3,478.00	3,479.00	3,480.00
Crude protein (%)	18.72	18.74	18.82	18.86
Crude fat (%)	5.75	5.75	5.75	5.76
SID lysine (%)	1.45	1.45	1.45	1.45
SID methionine (%)	0.60	0.60	0.61	0.61
SID threonine (%)	0.94	0.94	0.94	0.95
Total calcium (%)	0.62	0.64	0.62	0.61
Total phosphorus (%)	0.60	0.60	0.60	0.60
Chitin (%)^5)^	0.00	0.06	0.13	0.25

1) Control: corn-soybean meal-based diet, BSF25: corn-soybean meal based diet with 25% of plasma protein replaced by black soldier fly larvae, BSF50: corn-soybean meal based diet with 50% of plasma protein replaced by black soldier fly larvae, BSF100: corn-soybean meal based diet with 100% of plasma protein replaced by black soldier fly larvae.

2) Contents of vitamins provided per kg of complete diet: vitamin A, 10,000 IU; vitamin D3, 2,000 IU; vitamin E, 60 IU; vitamin K, 3.5 mg; vitamin B2, 8 mg; vitamin B6, 2 mg; vitamin B12, 35 μg; pantothenic acid, 25 mg; biotin, 100 μg; niacin, 50 mg; folic acid 3.1 mg; thiamine, 1.5 mg.

3) Contents of minerals provided per kg of complete diet: Fe, 100 mg; Mn, 50 mg; Zn, 50 mg; Cu, 80 mg; Se, 400 μg; I, 1 mg.

4) Lab analysis values.

5) Calculated values based on the chitin content of BSF (8.42%).

**Table 4:** Effects of different levels of black soldier fly larvae on growth performance in weaning pigs

Criteria	Treatment^1)^				SEM	p-value		
	
	Control	BSF25	BSF50	BSF100		ANOVA	Lin	Quad
Body weight (kg)								
Initial	8.12				-	-	-	-
2 wk	11.39^ab^	11.55^a^	11.17^ab^	10.84^b^	0.221	0.01	0.30	0.78
4 wk	16.22	16.11	15.90	15.51	0.252	0.32	0.32	0.93
ADG (g)								
0–2 wk	234.07	244.97	217.88	194.22	8.023	0.14	0.03	0.55
2–4 wk	343.99	325.75	337.60	333.40	6.872	0.87	0.79	0.70
0–4 wk	289.03	285.36	277.73	263.80	5.151	0.32	0.07	0.87
ADFI (g)								
0–2 wk	348.67	352.17	340.50	324.17	6.842	0.29	0.16	0.69
2–4 wk	508.25	488.57	473.16	453.28	15.341	0.53	0.22	0.83
0–4 wk	428.42	420.36	406.74	388.65	10.735	0.44	0.19	0.98
G:F ratio								
0–2 wk	0.67	0.70	0.63	0.59	0.022	0.12	0.03	0.64
2–4 wk	0.68	0.68	0.73	0.75	0.021	0.59	0.22	1.00
0–4 wk	0.68	0.69	0.69	0.68	0.015	0.15	0.87	0.71

1) Control: corn-soybean meal-based diet, BSF25: corn-soybean meal based diet with 25% of plasma protein replaced by black soldier fly larvae, BSF50: corn-soybean meal based diet with 50% of plasma protein replaced by black soldier fly larvae, BSF100: corn-soybean meal based diet with 100% of plasma protein replaced by black soldier fly larvae.

a,b Means with different superscripts in the same row significantly differ (p<0.05).

SEM, standard error of means; ANOVA, analysis of variance; Lin, linear; Quad, quadratic; ADG, average daily gain; ADFI, average daily feed intake; G:F, gain to feed.

**Table 5:** Effects of different levels of black soldier fly larvae on blood profiles in weaning pigs

Criteria	Treatment^1)^				SEM	p-value		
	
	Control	BSF25	BSF50	BSF100		ANOVA	Lin	Quad
Albumin (g/dL)								
Initial	4.17				-	-	-	-
2 wk	2.78	2.64	2.54	2.58	0.070	0.67	0.36	0.42
4 wk	3.06^a^	3.06^a^	2.40^b^	2.48^b^	0.085	0.01	0.01	0.07
Creatinine (mg/dL)								
Initial	1.04				-	-	-	-
2 wk	0.85	0.75	0.73	0.81	0.032	0.57	0.79	0.18
4 wk	0.81	0.86	0.80	0.84	0.020	0.67	0.91	0.96
Glucose (mg/dL)								
Initial	135.8				-	-	-	-
2 wk	87.6	89.4	81.4	84.2	1.921	0.49	0.37	0.61
4 wk	99.6	95.6	88.6	91.4	2.880	0.59	0.31	0.40
Total protein (g/dL)								
Initial	4.93				-	-	-	-
2 wk	4.60	5.26	4.60	4.50	0.178	0.44	0.51	0.49
4 wk	5.02	5.32	5.06	4.90	0.089	0.42	0.37	0.35
Globulin (g/dL)								
Initial	0.77				-	-	-	-
2 wk	1.82	2.62	2.06	1.92	0.215	0.59	0.81	0.41
4 wk	1.96	2.26	2.66	2.42	0.103	0.10	0.09	0.07
Urea (mg/dL)								
Initial	11.43				-	-	-	-
2 wk	14.24	16.74	16.68	19.90	1.204	0.46	0.13	0.97
4 wk	22.41	19.20	21.81	17.70	0.945	0.26	0.14	0.80
Total cholesterol (mg/dL)								
Initial	67.50				-	-	-	-
2 wk	70.2	65.41	75.00	72.24	1.753	0.27	0.37	0.83
4 wk	62.00	61.20	74.60	69.41	2.465	0.17	0.15	0.31
HDL cholesterol (mg/dL)								
Initial	67.67				-	-	-	-
2 wk	31.00^A^	25.60^B^	30.00^AB^	32.20^A^	0.960	0.07	0.21	0.11
4 wk	24.40	24.80	27.60	30.60	1.101	0.16	0.03	0.88
LDL cholesterol (mg/dL)								
Initial	108.70				-	-	-	-
2 wk	38.6	38.2	43.8	39.4	1.351	0.46	0.68	0.30
4 wk	38.2	36.4	47.0	40.2	1.800	0.17	0.43	0.22

1) Control: corn-soybean meal-based diet, BSF25: corn-soybean meal based diet with 25% of plasma protein replaced by black soldier fly larvae, BSF50: corn-soybean meal based diet with 50% of plasma protein replaced by black soldier fly larvae, BSF100: corn-soybean meal based diet with 100% of plasma protein replaced by black soldier fly larvae.

A,B Means with different superscripts in the same row significantly differ (p<0.05).

a,b Means with different superscripts in the same row significantly differ (p<0.05).

SEM, standard error of means; ANOVA, analysis of variance; Lin, linear; Quad, quadratic; HDL, high-density lipoprotein; LDL, low-density lipoprotein.

**Table 6:** Effects of different levels of black soldier fly larvae on immune response in weaning pigs

Criteria	Treatment^1)^				SEM		p-value	
	
	Control	BSF25	BSF50	BSF100		ANOVA	Lin	Quad
IgA (mg/mL)								
Initial	0.56							
2 wk	0.66	0.74	0.63	0.71	0.049	0.45	0.38	0.76
4 wk	0.95	0.90	1.02	0.86	0.069	0.51	0.24	0.30
IgG (mg/mL)								
Initial	5.76				-	-	-	-
2 wk	6.35	6.64	7.03	6.85	0.292	0.24	0.74	0.35
4 wk	7.03^ab^	7.24^a^	6.84^ab^	6.39^b^	0.518	0.07	0.42	0.06

1) Control: corn-soybean meal-based diet, BSF25: corn-soybean meal based diet with 25% of plasma protein replaced by black soldier fly larvae, BSF50: corn-soybean meal based diet with 50% of plasma protein replaced by black soldier fly larvae, BSF100: corn-soybean meal based diet with 100% of plasma protein replaced by black soldier fly larvae.

a,b Means with different superscripts in the same row significantly differ (p<0.05).

SEM, standard error of means; ANOVA, analysis of variance; Lin, linear; Quad, quadratic.

**Table 7:** Effects of different levels of black soldier fly larvae on diarrhea incidence in weaning pigs

Criteria	Treatment^1)^				SEM		p-value	
	
	Control	BSF25	BSF50	BSF100		ANOVA	Lin	Quad
Diarrhea incidence								
2 wk	1.15	1.03	1.25	1.22	0.081	0.69	0.61	0.70
4 wk	0.85	0.89	0.85	0.84	0.022	0.16	0.50	0.20
0–4 wk	1.00	0.96	1.05	1.03	0.474	0.47	0.41	0.36

1) Control: corn-soybean meal-based diet, BSF25: corn-soybean meal based diet with 25% of plasma protein replaced by black soldier fly larvae, BSF50: corn-soybean meal based diet with 50% of plasma protein replaced by black soldier fly larvae, BSF100: corn-soybean meal based diet with 100% of plasma protein replaced by black soldier fly larvae.

SEM, standard error of means; ANOVA, analysis of variance; Lin, linear; Quad, quadratic.

## References

[1] Lassaletta L, Estellés F, Beusen AHW (2019). Future global pig production systems according to the shared socioeconomic pathways. Sci Total Environ.

[2] Organization for Economic Co-operation and Development (OECD), Food and Agriculture Organization of the United Nations (FAO) (2023). OECD-FAO agricultural outlook.

[3] Alltech (2023). Agri-food outlook shares global feed production survey data and influential trends in agriculture.

[4] Food and Agriculture Organization of the United Nations (FAO) (2011). World livestock 2011 livestock in food security.

[5] Parisi G, Tulli F, Fortina R (2020). Protein hunger of the feed sector: the alternatives offered by the plant world. Ital J Anim Sci.

[6] van Huis A, Van Itterbeeck J, Klunder H (2013). Edible insects: future prospects for food and feed security.

[7] Kim B, Bang HT, Kim KH (2020). Evaluation of black soldier fly larvae oil as a dietary fat source in broiler chicken diets. J Anim Sci Technol.

[8] Hong J, Kim YY (2022). Insect as feed ingredients for pigs. Anim Biosci.

[9] Lu S, Taethaisong N, Meethip W (2022). Nutritional composition of black soldier fly larvae (Hermetia illucens L.) and its potential uses as alternative protein sources in animal diets: a review. Insects.

[10] El-Hack MEA, Shafi ME, Alghamdi WY (2020). Black soldier fly (Hermetia illucens) meal as a promising feed ingredient for poultry: a comprehensive review. Agriculture.

[11] Spranghers T, Michiels J, Vrancx J (2018). Gut antimicrobial effects and nutritional value of black soldier fly (Hermetia illucens L.) prepupae for weaned piglets. Anim Feed Sci Technol.

[12] Driemeyer H (2016). Evaluation of black soldier fly (Hermetia illucens) larvae as an alternative protein source in pig creep diets in relation to production, blood and manure microbiology parameters [master’s thesis].

[13] Boontiam W, Phaengphairee P, Hong J, Kim YY (2022). Full-fatted Hermetia illucens larva as a protein alternative: effects on weaning pig growth performance, gut health, and antioxidant status under poor sanitary conditions. J Appl Anim Res.

[14] Liu S, Wang J, Li L (2023). Endogenous chitinase might lead to differences in growth performance and intestinal health of piglets fed different levels of black soldier fly larva meal. Anim Nutr.

[15] Committee on Nutrient Requirements of Swine, National Research Council (2012). Nutrient requirements of swine.

[16] SAS Institute (2004). SAS user’s guide: statistics (version).

[17] Yu M, Li Z, Chen W, Rong T, Wang G, Ma X (2019). Hermetia illucens larvae as a potential dietary protein source altered the microbiota and modulated mucosal immune status in the colon of finishing pigs. J Anim Sci Biotechnol.

[18] Biasato I, Renna M, Gai F (2019). Partially defatted black soldier fly larva meal inclusion in piglet diets: effects on the growth performance, nutrient digestibility, blood profile, gut morphology and histological features. J Anim Sci Biotechnol.

[19] Crosbie M, Zhu C, Shoveller AK, Huber LA (2020). Standardized ileal digestible amino acids and net energy contents in full fat and defatted black soldier fly larvae meals (Hermetia illucens) fed to growing pigs. Transl Anim Sci.

[20] Khan JA, Guo X, Pichner R (2025). Evaluation of nutritional and techno-functional aspects of black soldier fly high-protein extracts in different developmental stages. animal.

[21] Zozo B, Wicht MM, Mshayisa VV, van Wyk J (2022). The nutritional quality and structural analysis of black soldier fly larvae flour before and after defatting. Insects.

[22] Dörper A, Veldkamp T, Dicke M (2021). Use of black soldier fly and house fly in feed to promote sustainable poultry production. J Insects Food Feed.

[23] Gasco L, Finke M, Van Huis A (2018). Can diets containing insects promote animal health? J. Insects Food Feed.

[24] Lestingi A (2024). Alternative and sustainable protein sources in pig diet: a review. Animals.

[25] Jin XH, Heo PS, Hong JS, Kim NJ, Kim YY (2016). Supplementation of dried mealworm (Tenebrio molitor larva) on growth performance, nutrient digestibility and blood profiles in weaning pigs. Asian-Australas J Anim Sci.

[26] Crosbie M, Zhu C, Karrow NA, Huber LA (2021). The effects of partially replacing animal protein sources with full fat black soldier fly larvae meal (Hermetia illucens) in nursery diets on growth performance, gut morphology, and immune response of pigs. Transl Anim Sci.

[27] Ipema AF, Gerrits WJJ, Bokkers EAM (2022). Assessing the effectiveness of providing live black soldier fly larvae (Hermetia illucens) to ease the weaning transition of piglets. Front Vet Sci.

[28] Blunt MC, Nicholson JP, Park GR (1998). Serum albumin and colloid osmotic pressure in survivors and nonsurvivors of prolonged critical illness. Anaesthesia.

[29] Tóthová C, Link R, Kyzeková P (2024). Serum protein electrophoretic pattern in piglets during the early postnatal period. Sci Rep.

[30] Sullivan DH, Johnson LE, Dennis RA (2011). The interrelationships among albumin, nutrient intake, and inflammation in elderly recuperative care patients. J Nutr Health Aging.

[31] Rye KA, Barter PJ (2014). Regulation of high-density lipoprotein metabolism. Circ Res.

[32] Chia SY, Tanga CM, Osuga IM (2019). Effect of dietary replacement of fishmeal by insect meal on growth performance, blood profiles and economics of growing pigs in Kenya. Animals.

[33] Van Hul M, Le Roy T, Prifti E (2020). From correlation to causality: the case of Subdoligranulum. Gut Microbes.

[34] Mair KH, Sedlak C, Käser T (2014). The porcine innate immune system: an update. Dev Comp Immunol.

[35] Wang Y, Zhao K, Li L (2023). A review of the immune activity of chitooligosaccharides. Food Sci Technol.

[36] Kawasaki K, Osafune T, Tamehira S (2021). Piglets can secrete acidic mammalian chitinase from the pre weaning stage. Sci Rep.

[37] Eggink KM, Lund I, Pedersen PB, Hansen BW, Dalsgaard J (2022). Biowaste and by-products as rearing substrates for black soldier fly (Hermetia illucens) larvae: effects on larval body composition and performance. PLOS ONE.

[38] Yousef M, Pichyangkura R, Soodvilai S, Chatsudthipong V, Muanprasat C (2012). Chitosan oligosaccharide as potential therapy of inflammatory bowel disease: therapeutic efficacy and possible mechanisms of action. Pharmacol Res.

[39] Boontiam W, Phaengphairee P, Hong J, Kim YY (2022). Full-fatted Hermetia illucens larva as a protein alternative: effects on weaning pig growth performance, gut health, and antioxidant status under poor sanitary conditions. J Appl Anim Res.

[40] Jin X, Yuan B, Liu M (2021). Dietary Hermetia illucens larvae replacement alleviates diarrhea and improves intestinal barrier function in weaned piglets challenged with enterotoxigenic Escherichia coli K88. Front Vet Sci.

[41] Phaengphairee P, Boontiam W, Wealleans A, Hong J, Kim YY (2023). Dietary supplementation with full-fat Hermetia illucens larvae and multi-probiotics, as a substitute for antibiotics, improves the growth performance, gut health, and antioxidative capacity of weaned pigs. BMC Vet Res.

